# Parkinson’s Disease: Biomarkers, Treatment, and Risk Factors

**DOI:** 10.3389/fnins.2018.00612

**Published:** 2018-08-30

**Authors:** Fatemeh N. Emamzadeh, Andrei Surguchov

**Affiliations:** ^1^Division of Biomedical and Life Sciences, Faculty of Health and Medicine, University of Lancaster, Lancaster, United Kingdom; ^2^Department of Neurology, Kansas University Medical Center, Kansas City, KS, United States

**Keywords:** Parkinson’s disease, biomarkers, α-synuclein, microRNAs, orexin

## Abstract

Parkinson’s disease (PD) is a progressive neurodegenerative disorder caused mainly by lack of dopamine in the brain. Dopamine is a neurotransmitter involved in movement, motivation, memory, and other functions; its level is decreased in PD brain as a result of dopaminergic cell death. Dopamine loss in PD brain is a cause of motor deficiency and, possibly, a reason of the cognitive deficit observed in some PD patients. PD is mostly not recognized in its early stage because of a long latency between the first damage to dopaminergic cells and the onset of clinical symptoms. Therefore, it is very important to find reliable molecular biomarkers that can distinguish PD from other conditions, monitor its progression, or give an indication of a positive response to a therapeutic intervention. PD biomarkers can be subdivided into four main types: clinical, imaging, biochemical, and genetic. For a long time protein biomarkers, dopamine metabolites, amino acids, etc. in blood, serum, cerebrospinal liquid (CSF) were considered the most promising. Among the candidate biomarkers that have been tested, various forms of α-synuclein (α-syn), i.e., soluble, aggregated, post-translationally modified, etc. were considered potentially the most efficient. However, the encouraging recent results suggest that microRNA-based analysis may bring considerable progress, especially if it is combined with α-syn data. Another promising analysis is the advanced metabolite profiling of body fluids, called “metabolomics” which may uncover metabolic fingerprints specific for various stages of PD. Conventional pharmacological treatment of PD is based on the replacement of dopamine using dopamine precursors (levodopa, L-DOPA, L-3,4 dihydroxyphenylalanine), dopamine agonists (amantadine, apomorphine) and MAO-B inhibitors (selegiline, rasagiline), which can be used alone or in combination with each other. Potential risk factors include environmental toxins, drugs, pesticides, brain microtrauma, focal cerebrovascular damage, and genomic defects. This review covers molecules that might act as the biomarkers of PD. Then, PD risk factors (including genetics and non-genetic factors) and PD treatment options are discussed.

## Introduction

Parkinson’s disease is the second most common neurodegenerative disease characterized by a progressive loss of dopaminergic neurons in the SN pars compacta. In PD, there is a long latency between the first damage to cells in at-risk nuclei of the nervous system, and the onset of clinical symptoms. The symptoms and signs of PD usually do not develop until 70–80% of dopaminergic neurons have already been lost (El-Agnaf et al., 2006). Thus, identifying patients in the period between the presumed onset of dopaminergic cell loss and the appearance of clinical parkinsonism may be of major importance for the development of effective neuroprotective treatment strategies (Berendse et al., 2001). Staining of LBs, the pathological hallmark of PD, to identify affected neurons throughout the nervous system, revealed six neuropathological stages of this disease based on the localization of the involved brain regions (Braak et al., 2003). Examination of brain samples from hundreds of PD patients revealed that the pathological process was relatively uniform. The pathology in the first stage begins in the lower medulla oblongata, specifically the dorsal motor nucleus of the vagal nerve, and the anterior olfactory structures. In stage 2, lesions in the dorsal motor nucleus worsen, inclusions develop in the lower raphe nuclei, and Lewy neurites can be observed in the locus ceruleus. In stage three the SN is affected. In stage four, lesions appear in the cortex, specifically in the temporal mesocortex. In stage five, the pathology appears in the adjoining temporal neocortical fields, while in stage six cortical involvement is clearly seen. Importantly, cognitive status correlates with the neuropathological stage (Braak et al., 2003). It is also essential to find reliable molecular biomarkers that can distinguish PD from other conditions, monitor its progression, or give an indication of a positive response to therapeutic intervention (Siderowf et al., 2018). α-Synuclein (α-Syn) aggregates are assumed to be harmful to dopaminergic neurons in the SN, and their formation may trigger the transmission of toxic α-syn from affected cells to other adjacent cells, resulting in a cascade of LBs formation and, subsequently, cell death (Angot and Brundin, 2009; Steiner et al., 2018). This promotes further dopaminergic cell loss caused by the spread of pathogenic forms of α-syn to neighboring cells (Luk et al., 2012). The transmission of α-syn between cells can be a normal pathway. However, in a stress condition, the aggregation of α-syn may be initiated within the receiver cells, where pre-aggregated α-syn acts as a ‘seed’ inducing more aggregation of soluble α-syn in a ‘prion-like’ fashion (Bernis et al., 2015). Furthermore, because α-syn aggregates are normally cleared by the proteasome system or by the lysosomes, any defect in clearance mechanisms could cause the spread of PD pathology as undigested toxic α-syn transmits to other cells. In accordance with this concept lysosomal inhibition enhances the amount of insoluble α-syn, leading to the elevated release of exosomes containing toxic α-syn (Luk et al., 2012). In healthy neurons unwanted proteins are cleared via an exosome mediated pathway, thus explaining why α-syn can be released from neurons in normal conditions, while any cellular or environmental problem that leads to higher α-syn secretion can be harmful to neurons and can be transmitted to other adjacent cells. There is a hypothesis that the accumulation of α-syn in PD patients begins in the enteric nervous system that is in nerves in the upper gastrointestinal tract (GI). α-Syn produced in GI is spread through vagus to the brain, suggesting a significant role of gut-brain axis in PD development (Liddle, 2018). Therefore, monitoring PD occurrence, diagnosis of early stage of this disease, ability to distinguish it from other parkinsonian syndromes, monitoring of its response to treatment and progression all require the identification of reliable biomarkers. After many years of disease PD can eventually evolve into PDD. The methods of diagnostics and distinctions between PD, PDD, and DLB are described in a recent comprehensive review (McKeith et al., 2017)

## Neurochemical Biomarkers

### Orexin

Orexin, also known as hypocretin, is a neuropeptide hormone expressed by a small number of neurons of the dorsolateral hypothalamus. Orexin is secreted by the lateral and posterior neurons of the hypothalamus. The hormone regulates many physiological functions, such as the sleep-wake cycle (Hagan et al., 1999), cardiovascular responses, heart rate, and hypertension (Imperatore et al., 2017). PD patients usually suffer from narcolepsy due to the loss of hypocretin neurons in the hypothalamus. The concentration of orexin A is lower in PD patients than in healthy individuals, and the level of orexin is related to the severity of the disease. The more severe is the disease, the higher loss of hypocretin neurons and the lower orexin levels in the CSF are observed (Fronczek et al., 2007). In the late stages of PD, decreased orexin levels may be responsible for daytime sleepiness (Wienecke et al., 2012). In narcolepsy of PD patients elevated levels of glial fibrillary acidic protein (GFAP) in the CSF seem to be causative for the reduction of orexin levels (Takahashi et al., 2015) pointing to GFAP as another potential biomarker. GFAP is an intermediate filament protein of the cytoskeleton that is expressed mostly in astrocytes. Hypophosphorylation and overexpression of GFAP often occur in PD patients, suggesting that these alterations in astrocytes are associated with the pathogenesis of PD (Clairembault et al., 2014). Astrocytes may be involved in the progression of PD by the production of pro-inflammatory cytokines that damage dopaminergic neurons (Rappold and Tieu, 2010). Therefore, PD can be identified by elevated levels of GFAP as an astroglial marker.

### 8-Hydroxy-2′-Deoxyguanosine

Reactive oxygen species (ROS) species (such as O_2_-, H_2_O_2_, and⋅OH) can damage biological molecules basically through irreversible reactions causing degenerative processes associated with aging. One of the DNA lesions caused by ROS is an oxidized form of 8-hydroxyguanine (8-OHG) known as 8-OHdG which can be used as a biomarker of DNA damage (Shigenaga et al., 1989). Likewise, an increase in 8-OHdG serum levels has been measured in PD patients compared to the normal individuals. CSF 8-OHdG levels are also higher in PD patients than in normal cases, but the difference is not as significant as serum levels. Therefore, 8-OHdG could be developed as a potential biomarker for PD (Kikuchi et al., 2002). It has been shown in the rat model that PD stage is directly related to urinary 8-OHdG level, suggesting that it can be used as a severity biomarker for PD (Kikuchi et al., 2011). However, it should be remembered that 8-OHdG is a marker of oxidative DNA damage, but not of progression of PD, so its specificity is limited (Simon et al., 2015).

### Peripheral Proteasomes and Caspase Activity

Proteasomes are large protein complexes responsible for degrading and elimination of unwanted and misfolded proteins and therefore are important for cell survival. Damaged proteins that are tagged with ubiquitin molecules by ubiquitin ligase, trigger the ATP-dependent proteolytic activity of the proteasome (Lodish et al., 2004). In PD, the accumulation of proteins within the neurons leads to the formation of pathological intracellular inclusions called LBs. Proteasome dysfunction may be involved in the formation of protein aggregates and associated with LBs (Bentea et al., 2017). In PD mutations disturbing proteasome activity may lead to the accumulation of aggregated α-syn (Shadrina et al., 2010; Ciechanover and Kwon, 2015). In some PD cases, mitochondrial deficiency causes the production of more ROS and higher α-syn oxidation leading to increased ATP-independent proteasomal activity and higher α-syn oligomerization. Depletion of ATP levels in this case inhibits 26S proteasome, but 20S complex still remains active and degrades oxidized α-syn (Martins-Branco et al., 2012). In advanced PD, the severity and duration of PD correlate with reduced proteasome 20S activity and increased caspase 3 activity. The activation of caspase and thus initiation of apoptosis is the main reason of proteasome 20S activity reduction. Therefore, these proteasome and caspase components may be also considered as potential PD biomarkers (Blandini et al., 2006).

### Dopamine, Dopamine Receptor, and Dopamine Transporter Activity

A catecholamine neurotransmitter dopamine is secreted by the SN, hypothalamus and some other regions of the brain. TH synthesizes the dopamine precursor (L-DOPA) that is converted to dopamine by L-aromatic amino acid decarboxylase (AADC). In the brain, dopamine is used as the precursor of noradrenaline (norepinephrine) and adrenaline (epinephrine). Loss of dopaminergic neurons in the midbrain and SN of PD brains leads to the reduction of dopamine levels (Obeso et al., 2008). The dopamine transporter (DAT) controls dopamine levels by facilitating its reuptake back to the cytosol. However, free dopamine is toxic for neurons, since its oxidation creates poisonous reactive quinones. Therefore, the vesicular monoamine transporter 2 (VMAT2) stores excess dopamine in vesicles. Thus, any change in dopamine or DAT levels may be an indicator of PD. Moreover, dopamine activates five types of receptors (D1R–D5R) and the severity of PD is related to the decreased expression of the dopamine type 3 receptor (D3R), leading to more severe symptoms because of reduced dopamine signals (Nagai et al., 1996). Therefore, D3R can be also considered as a potential biomarker for PD (Caronti et al., 2001).

Recent studies have point to 3-methoxy-4-hydroxyphenylglycol (MHPG) as a valuable biomarker to distinguish several forms of neurodegenerative diseases. This biogenic amine and norepinephrine’s metabolite passes the BBB, and analysis of its level in serum and CSF may be helpful to determine cognitive staging in PD, distinguish PD from non-PD controls, DLB versus AD, etc. (Vermeiren and De Deyn, 2017; van der Zee et al., 2018). This method is promising, since the locus coeruleus becomes affected in an earlier stage than the SN by α-syn.

In another recent article a preclinical phase of PD is identified by analysis of dopamine metabolites in CSF. Low CSF concentrations of 3,4-dihydroxyphenylacetic acid (DOPAC) and DOPA identify pre-clinical PD in at-risk healthy individuals (Goldstein et al., 2018).

Catecholamine neurons are not abundant in the nervous system, and their vulnerability in PD and related diseases (Goldstein et al., 2018) is not explained. A concept of autotoxicity assumes intrinsic cytotoxicity of catecholamines in cells in which it is produced. According to a recent theory, PD might develop when 3,4-dihydroxyphenylacetaldehyde (DOPAL) oligomerizes and aggregates α-syn, providing a link between synucleinopathy and catecholamine neuron loss in LBD (Goldstein et al., 2018).

According to the “catecholaldehyde hypothesis” for the pathogenesis of PD, long-term increased build-up of DOPAL, the catecholaldehyde metabolite of dopamine, causes or contributes to the eventual death of dopaminergic neurons. LBs, a neuropathological hallmark of PD, contain precipitated α-syn. Bases for the tendency of α-syn to precipitate in the cytoplasm of catecholaminergic neurons have also been mysterious. Since DOPAL potently oligomerizes and aggregates α-syn, the catecholaldehyde hypothesis provides a link between synucleinopathy and catecholamine neuron loss in Lewy body diseases. The concept developed here is that DOPAL and α-syn are nodes in a complex nexus of interacting homeostatic systems.

### α-Synuclein

α-Synuclein, which is found in an aggregated and fibrillar form, has attracted considerable attention as a potential molecular biomarker of PD. (Emamzadeh, 2016). Human α-syn is predominantly expressed in the brain in the neocortex, hippocampus, SN, thalamus and cerebellum, and is found in LBs (Surguchov, 2015). It is encoded by the SNCA gene that consists of six exons ranging in size from 42 to 1,110 bp (McLean et al., 2000; Yu et al., 2007). As noted above, the predominant form of α-syn is the full-length protein, but other shorter isoforms have been described. Importantly, C-terminal truncation of α-syn induces aggregation, suggesting that C-terminal modifications might be involved in the pathology of α-syn (Venda et al., 2010). Changes in the levels of α-syn have been reported in CSF and plasma of PD patients compared to control individuals (Hong et al., 2010). Recent evidence suggests that α-syn can be secreted into the extracellular space of the brain and spread the pathology of PD by propagating in a prion-like fashion (Masuda-Suzukake et al., 2013). This extracellular form of the protein can also be found in human body fluids, including blood and CSF (Mollenhauer et al., 2011). The gradual spread of α-syn pathology leads to a high concentration of extracellular α-syn that can potentially damage healthy neurons. Moreover, recent biomarker studies have shown changes in the level of α-syn in blood plasma from patients with PD (Li et al., 2007; Foulds et al., 2011), suggesting that α-syn might cross the BBB. Later, radiolabeled α-syn was traced in the bidirectional path from blood to the CNS and vice versa (Sui et al., 2014). Therefore, α-syn can be considered as a potential biomarker for PD.

### Apolipoprotein A1 (ApoA1)

ApoA1 is a 28 AA apolipoprotein with ∼28 kDa molecular weight which is the main constituent of HDL particles (Brewer et al., 1983). This apolipoprotein is synthesized mostly by the liver and the small intestine and is responsible for gathering extra cholesterol from cells. ApoA1 in cooperation with apoE participates in lipid transport in the brain (Emamzadeh, 2017). ApoA1 together with another apolipoprotein, apoE are responsible for lipid transportation in the brain. Lower levels of one isoform of apoA1 and tetranectin are reported in the CSF of PD patients, suggesting that apoA1 is a potential biomarker for PD (Wang et al., 2010; Swanson et al., 2015). ApoA1 cannot be secreted from neurons, but as the main component of HDL, it is required for cholesterol transportation to the brain. Therefore, it possibly passes through the BBB and contribute to the protective roles of HDL. In PD, lower level of apoA1 means less efficient HDL and reduced brain cholesterol homeostasis and function (Vitali et al., 2014).

### RNA-Based PD Biomarkers

Recent investigation of microRNAs (miRNAs) in PD point to their emerging role as potential PD biomarkers, especially due to their presence in CSF and peripheral circulation both free and in exosomes. miRNAs are small 21–24 nucleotide non-coding RNAs that regulate gene expression post-transcriptionally. Due to their ability to cross the BBB miRNAs have a high potential as convenient PD biomarkers. In a recent study Dos Santos et al. (2018) analyzed miRNA expression profile using next generation sequencing (NGS) in the CSF of early stage PD patients and controls. The authors have identified a miRNA-based biomarker panel for the early diagnosis of PD, including the five best ranking variables (Let-7f-5p, miR-125a-5p, miR-151a-3p, miR-27a-3p and miR-423-5p). The analysis showed high predictive value with 90% diagnostic sensitivity. Inclusion of α-syn in the analysis further improves robustness of a miRNA-based panel. Several other teams confirmed that microRNAs may be used as new PD biomarkers suggesting a breakthrough for novel diagnostic and therapeutic approaches to this disease (Arshad et al., 2017; Vitali et al., 2018). Several other biomarkers are under investigation in a number of Medical Centers, the information about which can be found on the website: https://clinicaltrials.gov/ct2/results?cond=Parkinson$+$Disease&term=biomarker&cntry=&state=&city=&dist=.

## Metabolite Profiling

Metabolic profiling of human tissues and/or biological fluids mirrors the complex interaction of genes, proteins and the environment of an individual. Proton (1H) and phosphorus (31P) magnetic resonance spectroscopy (MRS) are non-invasive imaging techniques that have been used to the study metabolites involved in energy metabolism, including ATP, lactate, creatine and other low molecular weight metabolite (Havelund et al., 2017). Significant alterations in metabolites have been described in PD patients and in animal models, including rise of lactate in striatum (Henchcliffe et al., 2008) and decrease of *N*-acetylaspartate/creatine ratios in advanced PD (Seraji-Bozorgzad et al., 2015). Alterations in alanine, branched-chain amino acids and fatty acid metabolism point to mitochondrial dysfunction in PD (Havelund et al., 2017).

## Neuroimaging Biomarkers

Today’s technology is able to detect brain’s abnormalities in PD patients using imaging techniques, such as transcranial B-mode sonography (TCS), susceptibility-weighted imaging (SWI), diffusion weighted imaging (DWI) (Chung et al., 2009), positron emission tomography (PET) scan and, single-photon emission computed tomography (SPECT) scan.

### Transcranial B-Mode Sonography (TCS)

Transcranial B-mode sonography monitors the blood flow velocity of brain’s vessels by measuring the frequency of ultrasounds waves and their echoes. This inexpensive and reliable method shows the higher echogenicity of the SN in PD brains compared to normal group that possibly occurs due to increased iron and gliosis levels in SN of PD patients (Skoloudík et al., 2014). The increased iron level in PD can be due to either alternation or malfunction of the BBB. In PD the increase in the number of iron-transferring receptors including both transferrin receptors of BBB and iron-binding receptors of neurons can lead to accumulation of iron in SN (Hare et al., 2013).

### Magnetic Resonance Imaging (MRI)

Diffusion weighted imaging is a form of MRI that measures the rate of water diffusion through a tissue to determine the structural details of that tissue. The higher measured diffusivity means the greater mobility of water molecules that can be due to the death of cells and the reduction of the region volume. This technique can differentiate PD from multiple system atrophy (MSA) in the early stage, while the clinical symptoms of these disorders are similar. In particular, the higher diffusivity of water in middle cerebellar peduncles in MSA patients in comparison to PD patients has been reported using DWI (Chung et al., 2009). DWI can also differentiate patients with progressive supranuclear palsy (PSP) from PD patients by detecting abnormalities in basal ganglia (Seppi et al., 2003). More traditional MRI methods, such as high-resolution 3-Tesla T1-weighted MRI can also detect the reduced volume of caudate and putamen in PD patients in compared to controls (Saeed et al., 2017).

### Single-Photon Emission Computed Tomography (SPECT) Scan

Both PET and SPECT scans can detect the early onset of PD and loss of dopaminergic neurons using radiotracers and computer techniques to generate 3D images. The majority of radiotracers are non-invasive radiopharmaceuticals with a short lifetime that usually decay soon after the imaging is complete. Moreover, the 3D images of PET and SPECT scans reveal function of an organ, whereas MRI can only monitor the anatomy and structure (Histed et al., 2012).

The SPECT scan radiotracers have a longer life-time in comparison to PET radiotracers. They mostly are ^123^iodine (^123^I) and ^99m^technetium (^99m^Tc) that emit gamma rays. The DAT-SPECT imaging can monitor degeneration of presynaptic terminals in dopaminergic neurons by visualizing DAT quantity. This method is a good way to diagnose reduction of DAT in the brain, and importantly it can’t distinguish PD and other Parkinsonian Syndromes. The DAT gamma-emitting ligands, such as ^123^I-iometopane (^123^I-β-CIT), ^123^I-ioflupane (^123^I-FP-CIT), ^123^I-altropane (^123^I-IPT) are the most common DAT-density SPECT tracers. These ligands are derivatives of tropane and dopamine reuptake inhibitors that target DAT (Wang et al., 2012; Brooks, 2016).

Single-photon emission computed tomography also employs dopamine D2 receptor radioligands that are dopamine antagonists. They include ^123^I-iodobenzamide (^123^I-IBZM) (Reiche et al., 1995), ^123^I-IBF (Sasaki et al., 2003), and ^123^I-epidepride (Pirker et al., 1997). ^123^I-2′-iodospiperone (2′-ISP) is also used in some studies to monitor D2 dopamine receptors. Although this radiotracer can distinguish between PD and other form of parkinsonism due to the pattern of its uptake in basal ganglia, it produces a high imaging background and needs to be modified to improve its performance (Yonekura et al., 1995).

The number of vesicular acetylcholine transporter (VAChT) can be monitored by SPECT radiotracers as an approach for PD early diagnosis. Acetylcholine (ACh) is a neurotransmitter that is in balance with dopamine in healthy people. The death of dopaminergic neurons and reduction of dopamine levels in PD are associated with increased Ach. ^123^I-iodobenzovesamicol (^123^I-IBVM) binds to VAChT and reveals the density of acetylcholine containing vesicles. Reduction of VAChT in parietal and occipital lobes in PD patients without dementia and reduced VAChT in all lobes of the cerebral cortex in PD patients with dementia has been established by this SPECT radiotracer (Niethammer et al., 2012).

^123^I-metaiodobenzylguanidine (^123^I-MIBG) is another radiotracer that can distinguish between PD and MSA (Goldstein, 2001). The heart-to-mediastinum ration of ^123^I-MIBG uptake is impaired in idiopathic PD patients, but not in patients with MSA. PET/CT scanning of these PD patients illustrated also decreased FP-CIT striatal uptake (Oh et al., 2015). MIBG scintigraphy allows to distinguish not only between PD versus MSA, but also between PD and DLB (Goldstein, 2001, 2013).

### Positron Emission Tomography (PET) Scan

The PET scan radiotracers emit electron anti-particles (positrons) that are positively charged with the same mass as an electron. The presence of presynaptic DAT in dopaminergic neurons of striatum and SN can be assessed with ^18^F and/or ^11^C radiolabeled dopamine analogs. These DAT radioligands include ^18^F-dopamine (^18^F-dopa) (Ibrahim et al., 2016), ^18^F-FE-PE2I (Fazio et al., 2015), ^18^F-β-CFT (Rinne et al., 1999), ^18^F-LBT999 (Arlicot et al., 2017), and^11^C-methylphenidate. The VMAT2 quantification is also possible by using either ^11^C or ^18^F radiolabeled dihydrotetrabenazine (DTBZ) (Tong et al., 2008; Lin et al., 2013). Because of dopaminergic cell loss and subsequent loss of VMAT2, the PET signal of radiolabeled DTBZ is lower in PD patients than in controls. Both DAT and VMAT2 radioligands can detect the early signs of dopaminergic damage, although PD may not be differentiated from atypical Parkinsonism with dopaminergic dysfunction. ^11^C-MP4A is another PET radiotracer that monitors the level of acetylcholinesterase (AChE) activity. AChE hydrolyses deactivates Ach and terminates the signal. Impairment of cholinergic system and reduction of cortical AChE has been assessed by ^11^C-MP4A-PET scan. AChE activity reduces more in PDD than in PD, indicating that cholinergic dysfunction is correlated with dementia in PD (Bohnen et al., 2006).

## Parkinson’s Disease Treatment

The development of neuroprotective drugs for PD is an important unmet medical need, since this disease progressively impair the patients’ quality of life and functionality in activities of daily living. The identification of new therapeutic targets is therefore of great importance. Although different medications and therapies for controlling PD symptoms are currently available, no cure for PD exists. The development of treatments for PD, based on patients’ symptoms and needs, vary from different medications to rehabilitation or even surgery. PD includes different clinical entities observed in several studies investigating the existence of PD subtypes. A cluster analysis permits to identify distinct PD subtypes according to the relevance of both motor and non-motor symptoms and select therapeutic approach according to cluster symptoms presentation (Lauretani et al., 2014).

## Medications

The most common therapy for PD includes different commercially available medications that treat the lack of dopamine in the SN. These medications can temporarily alleviate PD symptoms in different ways by enhancing dopamine level, mimicking the role of dopamine or inhibiting dopamine oxidative metabolism, which leads to the generation of reactive oxygen species (ROS) (Goldenberg, 2008). Formation of protein aggregates that lead to neuronal cell death is another important target for PD treatment. Among different PD medications, levodopa (L-dopa, L-3,4-dihydroxyphenylalanine) is an effective drug. Levodopa is the immediate metabolic precursor of dopamine which is produced by TH from L-tyrosine. In the dopaminergic neurons, dopa-decarboxylase converts levodopa into dopamine. The orally taken levodopa can be decarboxylated in peripheral sites before reaching the CNS. Therefore, levodopa is available in combination with carbidopa or benserazide that are peripheral inhibitors of Dopa decarboxylase, but do not pass through the BBB. Unchanged levodopa in the presence of decarboxylase peripheral inhibitors can penetrate into the CNS and is used as a precursor of dopamine (Goldenberg, 2008).

Levodopa, is more efficiently transformed into DA after vesicle storage by the serotonergic neurons, rather than the dopaminergic ones of the nigrostriatal system. Since the serotonergic distribution throughout the brain is very different than the dopaminergic one, this causes the well-known side effects of L-DOPA therapy and reduces its efficiency as a drug (De Deurwaerdère et al., 2017).

Sinemet was the first brand of carbidopa/levodopa combination in the pharmaceutical market (Scriabine, 1999). Rytary and duopa are two newly approved medications for PD by the Food and Drug Administration (FDA). Xadago (safinamide) is recently approved medication for PD patients who do not benefit from levodopa/carbidopa. Rytary is manufactured by Impax Laboratories as an oral capsule containing carbidopa-levodopa together with entacapone to prolong its effects. Doupa produced by AbbVie Company is used for the treatment of motor fluctuations in advanced PD. Doupa is an enteral gel made of levodopa-carbidopa that is pumped to patient intestines (Olanow et al., 2014).

A group of dopamine agonists are agents that bind to the dopaminergic post-synaptic receptors and trigger the same signal as dopamine itself. This group include pergolide, pramipexole dihydrochloride, ropinirole hydrochloride, rotigotine, and apomorphine hydrochloride (Jankovic and Aguilar, 2008).

Inhibitors of MAOB – selegiline and rasagiline are also available as PD medications. Monoamine oxidase isoforms, including MAOA and MAOB, located on the outer membrane of mitochondria are involved in the oxidative deamination of biogenic amines, such as neurotransmitters and xenobiotic amines, e.g., 1-methyl-4-phenyl-1,2,3,6-tetrahydropyridine (MPTP) (Bach et al., 1988). The MAOs have a significant effect on the course of PD, because they are involved in the metabolism of dopamine. Oxidative metabolism of dopamine in the dopaminergic cells of SN by MAOs leads to ROS generation, oxidative damage and cell death (Reiter, 1995). Moreover, MAOB catalyzes the conversion of MPTP into 1-methyl-4-phenylpyridine (MPP+) which is responsible for parkinsonism in intravenous drug users (Langston, 1996). Selegiline and rasagiline can protect neurons against oxidative damage induced by dopamine metabolites diminishing MAOB activity. Moreover, several substances, such as entacapone and tolcapone that inhibit catechol-*o*-methyl transferase (COMT) are available as alternative PD medications. These two medications block the conversion of levodopa into methylated levodopa. Therefore, inhibition of COMT activity by these medications can extend the existence of functional levodopa preventing its degradation (Goldenberg, 2008).

Additionally, some other chemicals can be considered as potential PD medicines. Rapamycin can be a useful treatment for PD as an up-regulator of autophagy. Rapamycin induces autophagy in cells by inhibition of a specific kinase activity called mTOR. Autophagy is a potential target for PD treatment, since it initiates the clearance of protein aggregates and inhibits apoptosis (Hochfeld et al., 2013). Adenosine A2A receptor antagonists, such as caffeine also reduces the risk of PD. Transgenic mice with mutant α-syn and deleted adenosine A2A receptor genes are protected against PD. Thus, A2A receptor antagonists are potential candidates for prevention and treatment of PD (Kachroo and Schwarzschild, 2012).

Formation of protein aggregates that leads to neuronal cell death is a promising target for PD treatment. In January 2015, Neuropore Therapies has announced phase I clinical trial of a new drug, NPT200-11 (UCB-1332) that inhibits oligomerization of α-syn (Oertel, 2017). Another potential drug modulating the aggregation of α-syn blocking or reducing the conversion of monomers to oligomers or later on to fibrils is ANLE138b (Levin et al., 2014). Several promising compounds are under development and/or in preclinical testing that may enhance autophagy of α-syn. Recent screening of compounds protecting cells from α-syn induced neurodegeneration identified a non-selective phosphodiesterase (PDE) inhibitor dipyridamole. Importantly, PDE1 inhibition also protects dopaminergic neurons from α-syn induced degeneration in mouse SN. PDE inhibitors are currently at preclinical testing (Höllerhage et al., 2017). Another antiaggregation compound – a natural product squalamine displaces α-syn from the surfaces of lipid vesicles, thereby blocking the first steps in its aggregation process. Furthermore, squalamine suppresses the toxicity of α-syn oligomers by inhibiting their interactions with lipid membranes (Perni et al., 2017).

## Surgery

Deep brain stimulation therapy is rarely used for certain types of brain-related disorders including PD, dystonia, obsessive-compulsive disorder and treatment resistant depression (Herrington et al., 2016). When PD symptoms are very severe and medications cannot moderate them, surgery and DBS can be considered as the final options for the treatment. It involves sending electrical impulses to certain parts of the brain (usually SN or globus pallidus, which communicate with the SN) by a neurostimulator device that is a brain implant known as a ‘brain pacemaker.’ The target area of DBS is usually the subthalamic nucleus (STN). The stimulation of the dorsolateral STN border alongside the surgery can improve its efficiency (Herzog et al., 2004). Later it was found that stimulation of caudal zona incerta (cZI) can be more effective with fewer complications after the surgery (Plaha et al., 2006). Stimulation of neurons may also lead to neurogenesis and neuroplasticity and thus can improve for a long time motor problems, such as dyskinesia and tremor, and all other levodopa-responsive symptoms, for a long time. However, there are two problems with DBS, namely a 3- to 6-month waiting period required for optimal results and the possibility of brain infection (Bronstein et al., 2011).

## Gene Therapy

The development of gene therapy of PD has made a major progress in a recent decade. Advanced PD does not give a good response to levodopa therapy. Broaden loss of dopaminergic neurons is accompanied by reduction in aromatic amino acid decarboxylase (AADC) levels that converts L-DOPA to dopamine. After successful preclinical studies, adeno-associated viral vectors carrying human AADC gene are recently delivered into putaminal neurons and subthalamic nucleus of PD patients. In this method, sufficient amount of dopamine production can be controlled by taking adequate levodopa dose. Orally taken levodopa can be converted into dopamine by AADC and sooth PD symptoms (Muramatsu et al., 2010). Safety and efficiency of the method have been proven over 4 years by annually PET imaging from patients who received specific dosages of AAV2-hAADC (Mittermeyer et al., 2012). Another target for gene therapy in PD is glutamic acid decarboxylase (GAD) that facilitate production of GABA in GABA-ergic neurons in the subthalamic nucleus. GABA as an inhibitory neurotransmitter regulates muscle tone and improve motor functions (Watanabe et al., 2002). Lack of dopamine in PD causes activation of the subthalamic nucleus and unnecessary muscular responses that GABA by its inhibitory effect can significantly improve. This method can be compared with DBS that by sending electric shocks to subthalamic nucleus reduces its hyperactivity and improve motor impairment (Coune et al., 2012). The human trial of this method was performed by bilateral injection of AAV2-GAD in the subthalamic nucleus of patients with advanced PD. This approach has shown safety and efficiency, although it needs more investigation to be considered as a treatment (LeWitt et al., 2011). Therapeutic effect of glial cell line-derived neurotrophic factor (GDNF) on neuronal function in non-human models of PD encouraged scientists to inject it directly into putamen of PD patients. This surgery helped patients with improved movement, reduced dyskinesia and increased dopamine storage in the putamen (Gill et al., 2003). Therefore, GDNF is a possible option for gene therapy. Neurturin gene is another candidate for PD gene therapy. Neurturin is a neurotrophic factor important for survival and differentiation of dopaminergic neurons (Lin et al., 1993). The human trial of this factor was conducted by bilateral injection of vector AAV2-neurturin (CERE-120) into the putamen biomarkers patients with advanced PD (Bartus et al., 2013). Injected neurturin cannot spread into SN and play its therapeutic role because of vast axonal transport defects in PD patients. Another consequence of this method may be the accumulation of α-syn that leads to downregulation of neurturin expression (Bartus et al., 2014).

## Non-Genetic Risk Factors of Parkinson Disease

Only 10–15% of PD cases are early onset familial PD, while the remaining cases are idiopathic pointing to an important role of non-genetic and environmental factors in PD pathogenesis. Exposure to environmental toxins can cause dopaminergic cell death. The accumulation of heavy metals in the SN enhances the risk of developing PD. The effect of exposure to heavy metals could increase oxidative stress in dopaminergic cells, leading to PD. MAO in the presence of oxygen can mediate dopamine oxidation *in vitro* into 3,4-dihydroxylphenyl-acetaldehyde (DOPAL). DOPAL initiates oligomerization of α-syn into non-fibrillar, SDS-resistant aggregates (Burke, 2003). However, another study has revealed that inhibition of MAO to stop the production of DOPAL is not sufficient to reduce oligomerization of α-syn (Burke, 2003). The auto-oxidation of dopamine to dourmine quinone (DAQ) can also increase both formation and secretion of non-fibrillar α-syn oligomers, thus promoting pathogenic α-syn transmission to adjacent neurons and glia (Lee et al., 2011). Neuromelanin pigment is highly expressed in dopaminergic neurons, preventing oxidative stress related to the accumulation of cytosolic dopamine. On the other hand, dying neurons in PD brains release neuromelanin that activates neuroglia and triggers neuroinflammation (Zucca et al., 2014). Moreover, lipidated neuromelanin can interact with α-syn and trigger its aggregation into the insoluble complex in PD patients (Double and Halliday, 2006).

Genetic polymorphisms of cytochrome P450 2D6 (CYP2D6) – an enzyme involved in metabolizing environmental toxins, are also related to the development of PD. For example, CYP2D6 deficient metabolizers are two times higher at risk of developing PD if they are also exposed to pesticides. Therefore, sufficient levels of CYP2D6 activity are required for the metabolism of pesticides (e.g., organophosphates, atrazine), which are linked to the pathogenesis of PD (Elbaz and Tranchant, 2007).

Reactive oxygen species and thus oxidative stress is also known as a pathological factor in PD. NADPH oxidase-2 enzyme (NOX2) is a membrane-bound oxidase present primarily in phagocytes generating ROS in phagosomes to kill bacteria. NOX2-derived ROS also damages dopaminergic neurons. Dopaminergic neurons in NOX2-knockout mice start to degenerate faster than similar cells in wild-type controls after administration of MPTP (Brieger et al., 2012).

In 1956 two neurologists, Poskanzer and Schwab hypothesized that PD is related to influenza infection (Estupinan et al., 2013). They studied a group of PD patients and revealed that age of disease onset is shifted in the direction of an age group who were born before or during the influenza pandemic in 1918 and mostly had been infected by the flu virus. However, other studies showed that influenza infection can cause PD-like symptoms, but cannot increase the risk of developing PD (Estupinan et al., 2013). Another hypothesis proposes that Toxoplasma gondii, an intracellular parasite causing toxoplasmosis, may increase the risk of PD. The parasite in the brain infected those areas that are affected in PD, including basal ganglia (Miman et al., 2010), although other studies did not get a similar result (Mahami Oskouei et al., 2016).

## Genetic Forms and Genetic Risk Factors of PD

Although most cases of PD are idiopathic forms of the disease, about 15% of PD patients are recognized as having a first-degree family member with this disease. Recently, the genetic factors and gene loci involving in autosomal dominant and autosomal recessive forms of PD have been discovered due to advanced molecular genetics (Samii et al., 2004; Karimi-Moghadam et al., 2018) (**Tables 1, 2**). The mutations in several genes, including α-syn, LRRK2, PINK1, Parkin, DJ-1, VPS35 and GBA1 are linked to PD (Zeng et al., 2018). In addition to mutations in these genetic loci, polymorphisms, and trinucleotide repeats are recognized as PD genes, or susceptibility factors for PD (**Table 3**).

**Table 1:** Autosomal recessive and X-linked genes involved in Parkinson’s disease.

Inheritance pattern	Locus	Chr. location	Mutation site in	Involved protein
**Autosomal recessive**	PARK2/PARKN	6q25.2-q27	*Parkin*	Ubiquitin-protein ligase (Kitada et al., 1988)
	PARK6	1p36	*PINK1*	PTEN-induced putative kinase 1 (Valente et al., 2004)
	PARK7	1p36.23	*DJ1*	Oncogene DJ1 (van Duijn et al., 2001)
	PARK9	1p36	*ATP13A2*	Lysosomal type 5 P-type ATPase (Ramirez et al., 2006)
	PARK14	22q13.1	*PLA2G6*	Phospholipase A2 (Paisan-Ruiz et al., 2009; Shi et al., 2011)
	PARK15	22q12-q13	*FBXO7*	F-BOX only protein 7 (Paisan-Ruiz et al., 2009)
	PARK19	1p32	*DNAJC6*	Putative tyrosine-protein phosphatase auxilin (Koroglu et al., 2013)
	PARK20	21q22	*SYNJ1*	Synaptojanin-1 (Krebs et al., 2013)
**X-linked**	PARK12	Xq21-q25	*TAF1*	TFIID subunit 1 (Graeber and Muller, 1992)

**Table 2:** Autosomal dominant genes involved in Parkinson’s disease.

Inheritance pattern	Locus	Chr. location	Mutation site	Involved protein
**Autosomal dominant**	PARK1	4q21	*SNCA*	α-Syn (Xu et al., 2017)
	PARK3	2p13.2	*SPR*	Sepiapterin reductase in BH4 pathway (Karamohamed et al., 2003)
	PARK4	4q21	Triplication of *SNCA*	α-Syn (Singleton et al., 2003)
	PARK5	4p14	*UCHL1*	Ubiquitin C-terminal hydrolase (Leroy et al., 1998)
	PARK8	12q12	*LRRK2*	Leucine-reach repeat kinase 2 (Zimprich et al., 2004)
	PARK11	2q37	*GIGYF2*	GRB10-interacting GYF protein 2 (Lautier et al., 2008; Tan et al., 2009)
	PARK13	2p12	*HTRA2*	HTRA serine peptidase (Strauss et al., 2005; Lin et al., 2011)
	PARK16	1q32	Multiple independent sites?	Unknown (Satake et al., 2009; Li et al., 2011)
	PARK17	16p12.1-q12.1	*VPS35*	Vacuolar protein sorting 35 (Vilariño-Güell et al., 2011)
	PARK18	3q27	*EIF4G1*	Eukaryotic translation initiation factor 4 gamma 1 (Chartier-Harlin et al., 2011)
	PARK21	3q22	*DNAJC13*	DNAJ-domain-bearing protein (Appel-Cresswell et al., 2014; Vilariño-Güell et al., 2014)

**Table 3:** Susceptibility factors of Parkinson’s disease.

Susceptibility factors	Involved gene	Chr. location	Putative function	Phenotype
	*GBA*	1q21	Acid β-glucocerebrosidase	Gaucher disease (Pulkes et al., 2014)
	*MAPT*	17q21.1	Microtubule-associated protein tau	Supranuclear palsy, Dementia (Das et al., 2009)
	*MC1R*	16q24.3	Melanocyte-stimulating hormone receptor	Albinism (Tell-Marti et al., 2015)
	*ADH1C*	4q23	Alcohol dehydrogenase 1C	Alcohol dependence (Buervenich et al., 2005)
	*ADH4*	4q22	Alcohol dehydrogenase 4	Alcohol dependence (Buervenich et al., 2000)
	*HLA*	6p21.3	Major histocompatibility complex	Imamura et al., 2003
	*ATXN2*	12q24.1	Ataxin-2	Spinocerebellar ataxia 2 (Charles et al., 2007)
	*ATXN3*	14q21	Ataxin-3	Machado-Joseph disease (Gwinn-Hardy et al., 2001)
	*TBP*	6q27	TATA box-binding protein	Spinocerebellar ataxia 17 (Wu et al., 2004)
	*ATXN8OS*	13q21.33	Ataxin-8 opposite strand	Spinocerebellar ataxia 8 (Wu et al., 2004)
	*NR4A2*	2q24.1	Nuclear receptor subfamily 4 group A member 2 (transcription factor)	Le et al., 2003

## Epigenetic Risk Factors of PD

Epigenetics refer to chromatin alternations, including DNA methylation and histone post translational modifications that can alter gene expression without changes in DNA sequence. These modifications can be inherited, but environmental factors including nutritional, chemical and physical factors can also affect epigenetics (Surguchov et al., 2017). In sporadic form of PD involvement of environmental factors in initiation and progression of disease emerging an idea that epigenetic plays an important role in PD (Feng et al., 2015). One of the examples of epigenetic mechanism in PD is modification of α-syn gene (SNCA). SNCA has two CpG islands with the first one being located in exon 1 (CpG-1) and the second one (CpG-2) within intron 1. Transcription factors (TFs) GATA and ZSCAN21 bind to the intron 1 CpG-2 island and modulate transcription of α-syn. CpG-2 methylation prevents the binding of the TFs to SNCA and subsequently inhibits the overexpression of α-syn. Interestingly, binding of transcription factors to α-syn (Iwata et al., 2001) and another member of the synuclein family, γ-synuclein (Surgucheva and Surguchov, 2008), has been described, suggesting that synuclein family members are involved in various complex mechanisms of gene expression regulation. Therefore, their role in PD is not limited to the formation of toxic aggregates, but may be complemented by participation in regulatory processes.

Matsumoto et al. (2010) found that CpGs in SNCA were hypermethylated in controls, but not methylated in PD patients, suggesting that methylation was an epigenetic risk factor for PD that is related to the pathogenesis of α-syn. In PD, demethylated SNCA codes for a high amount of α-syn that may initiate aggregation and promote neurodegeneration. Additionally, the levels of nuclear DNA (cytosine-5′) methyltransferase 1 (DNMT1) in post-mortem PD brains is lower than in control brains. This methylase epigenetically suppresses gene expression by DNA methylation, and its reduced level in PD may be associated with SNCA hypomethylation.

Epigenetic modifications in other genes are also implicated in contributing to PD, including tumor necrosis factor α (TNF-α), PARK16, transmembrane glycoprotein NMB (GPNMB), and syntaxin-1B (STX1B) genes. Hypomethylation of the TNF-α promoter, hypermethylation of a CpG dinucleotide in synphilin-1, STX1B and hypermethylation of multiple CpG sites proximal to GPNMB are all described in PD (Yang et al., 2017). Moreover, the epigenetic changes in mitochondrial DNA (mtDNA) can also trigger PD. Several studies show the hypo-methylation and hyper-hydroxymethylation of mtDNA displacement loop (D-loop) in the SN of PD brains (Iacobazzi et al., 2013; Chuang et al., 2017).

## Conclusion

Pathology of PD is complex and include a combination of genetics, epigenetics and environmental factors. Current medical, pathological, and experimental data support the Braak hypothesis (Braak et al., 2003) of spatiotemporal spread of PD pathology involving α-syn propagation from the gastrointestinal and olfactory system via transsynaptic cell-to-cell transfer through the vegetative nervous systems to the CNS. Many non-motor symptoms, including sleep disorders, olfactory deficiency, hyposmia (reduced ability to smell and to detect odors), constipation and others may play an important role as successful, future diagnostic to target PD pathology and mechanisms during the initial stages of disease (Reichmann et al., 2016). Important contributing factors to PD are dysfunctions of mitochondria and endoplasmic reticulum, impairment of autophagy and endocytosis, and deregulation of immunity. Despite intensive investigation of PD mechanism the disease is still incurable. Future challenges for physicians and researchers working in the field of PD and similar disorders will be directed to the identification of biomarkers for an early diagnosis of these diseases at preclinical stage. New biomarkers should follow the progression of the disease and determine key metabolomic alterations to identify potential targets for intervention. Future tasks should be also directed to the development and application of preventive measures to stop or reduce disease progression at pre-symptomatic stages and the finding of novel antiparkinsonian drugs with specific neuroprotective effects on the dopaminergic system. In addition to conservative approach based on the identification of new protein biomarkers in blood, plasma and CSF, there is a growing hope that analysis of microRNAs may greatly contribute to early diagnosis of this disease. The development of induced pluripotent stem cell (iPSC) and genome editing technique open new hopes for the treatment of several human diseases, especially genetic disorders. For PD which is predominantly sporadic disease and a mix of several relatively rare genetic causes these methods gave the opportunity to develop new models and study phenotypes in both genetic and sporadic cases to piece together pathogenic pathways involving their gene products.

## Author Contributions

FE designed the article contents and wrote the original manuscript. AS revised and edited the manuscript, searched for additional related literature and discussed the writing with FE.

## Conflict of Interest Statement

The authors declare that the research was conducted in the absence of any commercial or financial relationships that could be construed as a potential conflict of interest.

## Abbreviations

8-OHdG: 8-hydroxy-2′-deoxyguanosine
BBB: blood–brain barrier
CSF: cerebrospinal fluid
DBS: deep brain stimulation
DLBs: dementia with Lewy bodies
GBA: acid β-glucocerebrosidase
HDLs: high density lipoproteins
LBs: Lewy bodies
LDLs: low density lipoproteins
L-dopa: levodopa
LRRK2: leucine-rich repeat kinase 2
MAOA: monoamine oxidase A
MAOB: monoamine oxidase B
MAPKs: mitogen-activated protein kinases
MAPT: microtubule-associated protein tau
MPTP: 1-methyl-4-phenyl-1,2,3,6 tetrahydropyridine
PD: Parkinson’s disease
PDD: Parkinson’s disease dementia
PEA: β-phenylethylamine
PINK1: PTEN-induced putative kinase 1
SN: substantia nigra
TBP: TATA box-binding protein
TH: tyrosine hydroxylase
VLDLs: very low density lipoproteins
